# Case Report: A severe drug-induced immune hemolytic anemia caused by vancomycin

**DOI:** 10.3389/fmed.2025.1686669

**Published:** 2025-11-21

**Authors:** Yangyi Xie, Zhongying Wang, Danfei Deng, Zhe Zhu, Zhen Huang, Jing Wang, Dong Xiang, Yong Yu

**Affiliations:** 1 Department of Blood Transfusion, Ningbo No.2 Hospital, Ningbo, China; 2 Blood Group Reference Laboratory, Shanghai Institute of Blood Transfusion, Shanghai Blood Center, Shanghai, China; 3 Department of Laboratory Medicine, Children’s Hospital of Shanghai, Shanghai, China

**Keywords:** drug-induced immune hemolytic anemia, vancomycin, hemoglobin, antibody, serological test

## Abstract

**Background:**

Drug-induced immune hemolytic anemia (DIIHA) is an adverse effect of some therapeutics, which is potentially life-threatening. A high priority is required in clinical treatment, since the number of medications known to induce DIIHA continues to expand. This study reports on a case of vancomycin-induced hemolysis observed in a patient in China.

**Case description:**

A 50-year-old man with negative irregular antibody detection tests developed hemolytic anemia on the eighth day after replacement and repair surgery of his left knee. The patient was treated with vancomycin-mixed bone cement during the surgery, as well as vancomycin intravenously in the postoperative period every day. On the eighth day after surgery, he developed a rapid decline in hemoglobin level, elevation of indirect bilirubin and lactate dehydrogenase levels in serum, and presence of hemoglobin in urine. Laboratory analysis revealed high-titer antibodies against vancomycin in the patient’s serum, which were mainly immunoglobulin G (IgG) antibodies, and demonstrated greater specificity for the blood group N antigen.

**Conclusion:**

This case, together with previous reports, underscores the importance of considering DIIHA in patients who exhibit unexplained decreases in hemoglobin levels following a new medication, especially antibiotics. A thorough examination of the patient’s medical history and the identification of specific drug-dependent antibodies through serological tests can provide crucial insights for diagnosing DIIHA.

## 1 Introduction

Drug-induced immune hemolytic anemia (DIIHA) remains a serious and unrecognized diagnosis, with an estimated annual incidence of one per million people, although this may be underestimated because only severe hemolytic events are typically reported (1). It can be mild or associated with acute, severe hemolytic anemia and even death. DIIHA is most commonly attributed to drug-dependent antibodies that can be detected only in the presence of the drug, which can bind to red blood cell (RBC) membrane proteins either covalently or non-covalently (2). DIIHA can also be associated with drug-independent antibodies, which do not need the drug to be present to obtain *in vitro* reactions. In these cases, the drug affects the immune system, causing the production of RBC autoantibodies, and the clinical and laboratory findings are identical to autoimmune hemolytic anemia, other than the remission associated with discontinuing the drug (2).

More than 150 drugs have been described to potentially induce immune hemolytic anemia (3). Historically, methyldopa and large doses of penicillin were the most frequent causes of DIIHA. Currently, the majority of cases are caused by ceftriaxone, other cephalosporins, piperacillin, or non-steroidal anti-inflammatory drugs (4). The primary therapeutic strategy involves identifying and discontinuing the offending drug. Serological tests are often required to confirm the diagnosis of DIIHA (5). Drug-dependent antibodies can be detected by testing with drug-treated RBCs or untreated RBCs in the presence of soluble drugs, because not all drug-dependent antibodies react in the same manner. Unfortunately, DIIHA caused by drug-independent antibodies is indistinguishable from autoimmune hemolytic anemia, as drug-independent antibodies cannot be ascertained or excluded by serological tests.

Vancomycin is an antibiotic commonly used in the treatment of severe Gram-positive bacterial infections and is a component of an empirical antibiotic regimen in total joint replacement (6). While DIIHA associated with vancomycin was previously considered rare (7), there are 56 cases of hemolytic anemia and 21 cases of hemolysis with vancomycin reported in VigiAccess, the WHO global database of adverse drug reactions, alongside bleeding cases linked to immune-mediated thrombocytopenia (8, 9). Yet, the underlying molecular mechanisms by which vancomycin induces DIIHA have not been well established.

In this study, we report a case of a 50-year-old man who experienced severe hemolytic anemia during intravenous vancomycin therapy after surgery for replacement and repair of the left knee. The serological tests detected vancomycin-dependent antibodies. Although intravenous vancomycin was promptly discontinued and blood transfusion was administered, the patient unfortunately died of cerebral infarction 1 day after drug withdrawal. This case highlights the importance of early recognition and immediate discontinuation of the causative drug, as well as the initiation of therapeutic interventions. This study was approved by the Ethics Committee of Ningbo No.2 Hospital. This case report was drafted in accordance with the CARE (Case Reports) guidelines (10).

## 2 Case description

**Figure 1:**
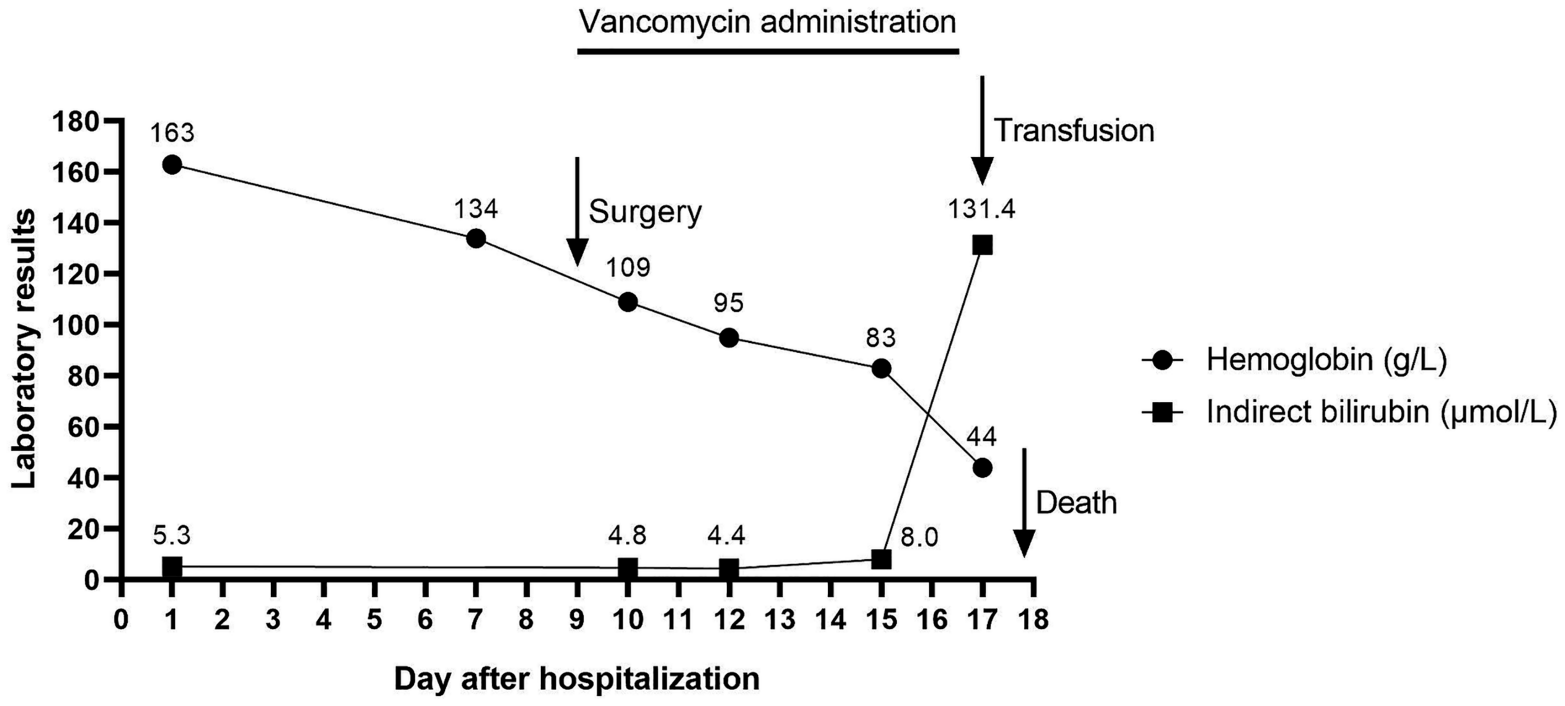
Patient’s hemoglobin and indirect bilirubin levels after hospital admission.

A 50-year-old man with infectious arthritis of the left knee was admitted to the hospital in December 2024, with comorbidities including diabetes, hypertension, renal insufficiency, hyperlipidemia, and a history of cerebral infarction. Although no cause of cerebral infarction was found in the patient’s past medical records at our hospital, his comorbidities, which were all risk factors for cerebral infarction, severely damaged the cerebrovasculature, making an early-onset ischemic stroke not only possible but highly likely. Accordingly, the patient received preoperative evaluations from the Neurology, Nephrology, and Endocrinology departments to optimize his condition for surgery. In March 2022, he presented with pain and limited mobility in the left knee joint, followed by arthroscopic left knee meniscus suture surgery. Nevertheless, the postoperative pain of the patient gradually aggravated, and he was diagnosed with a left knee joint infection and underwent arthroscopic debridement of the left knee joint in June 2022. Over the past several years, he experienced progressively limited mobility of the left knee joint and worsening pain. Therefore, he underwent total knee replacement and tibial insertion surgery during this hospitalization. During the surgery, the patient received vancomycin-mixed bone cement. The surgery went smoothly without the need for a blood transfusion. Postoperatively, the patient was conscious, and his vital signs were stable. He received routine postoperative care, including preventive anticoagulation with enoxaparin sodium, analgesic therapy with parecoxib sodium, as well as anti-infection treatment with intravenous vancomycin (1 g) every 12 h. However, on the eighth postoperative day, his hemoglobin level sharply decreased to 44 g/L, and an emergency blood transfusion was required (Figure 1). Laboratory tests showed elevated indirect bilirubin (131.4 μmol/L), elevated lactate dehydrogenase (1,080 U/L), elevated reticulocyte percentage (6.1%), no bacterial growth in blood cultures, and positive urinary occult blood. Hemolytic anemia was suspected.

### 2.1 Diagnosis based on serological tests

The patient’s blood type was identified as AB, Rh CCDee, and irregular antibody screening tests were negative at admission, consistent with previous results. Irregular antibodies, also known as unexpected antibodies, are blood group antibodies other than anti-A and anti-B, which typically arise following immune stimulation from events such as blood transfusions or pregnancy (11). Since these antibodies can cause transfusion reactions, irregular antibody screening is a critical step before blood transfusion. The patient had not received any blood transfusions from admission until that time, and his last RBC transfusion had been over 2 years ago. When we received his emergency blood sample, his RBCs were autoagglutinated, and the plasma was dark brown. The RBCs were washed three times with saline and then evaluated using direct antiglobulin tests (DATs). DATs were positive (2+) with anti-IgG and strongly positive (4+) with anti-C3. Interestingly, the antibody screening tests turned positive: I(1+), II(2+), III(±) (Table 1). The irregular antibody identification demonstrated possible anti-N (Table 2), while the patient’s MN blood type was identified as M-N+. 2-mercaptoethanol (2-ME) treatment, to dissociate immunoglobulin M (IgM) antibody molecules, did not alter reactivity, indicating the antibodies were IgG, which was also identified by flow cytometry. The anti-N titer was 4, tested using M-N+ screening cells. The acid eluate also demonstrated weak reactivity consistent with anti-N (Table 2), and the titer of anti-N was approximately 1. In that case, the patient was placed on an N antigen-negative RBC transfusion protocol. He received three units of RBCs in the evening of the eighth postoperative day. Each unit of RBCs, with additives, was approximately 150 mL, derived from 200 mL of whole blood. However, the efficacy of the blood transfusion could not be determined due to the patient’s death in the early morning of the following day (the ninth postoperative day). The patient’s fatal cerebral infarction and subsequent shock were highly likely to be direct consequences of the severe hemolytic anemia, since the destruction of RBCs would critically reduce oxygen delivery to vital organs, including the brain, and the release of pro-coagulant substances from the lysed RBCs would create a hypercoagulable state, significantly increasing the risk of thrombotic events such as cerebral infarction.

**Table 1:** Results of antibody screening tests.

Antibody screening cells	Rh-hr					Kidd		MNS				Duffy		Lewis		P	Agglutination intensity
	D	C	E	c	e	Jka	Jkb	M	N	S	s	Fya	Fyb	Lea	Leb	P1	The patient’s serum
I	+	0	+	+	0	0	+	+	+	+	+	+	+	0	+	0	1+
II	+	+	0	0	+	0	+	0	+	0	+	+	0	+	+	+	2+
III	+	+	+	+	+	+	0	+	0	0	+	+	0	0	+	+	w

The irregular antibody screening tests were performed using immediate spin techniques with antibody screening cells (Shanghai Hemo Pharmaceutical & Biological Co., Ltd). The test data were from three independent experiments. Agglutination intensity was graded from no agglutination (0) to strong agglutination (4+). w: weakly positive.

**Table 2:** Reaction results with panel cells for antibody identification.

Panel cells	D	C	E	c	e	K	k	M	N	S	s	P1	Lea	Leb	Fya	Fyb	Jka	Jkb	Dia	Kpa	Kpb	Lua	Lub	Agglutination intensity	
																								Serum	Eluate
1	+	+	0	0	+	+	0	+	+	0	+	+	0	+	0	+	+	+	0	0	+	0	+	1+	w
2	+	0	+	+	0	0	+	0	+	+	+	0	0	+	+	0	0	+	0	0	+	0	+	2+	1+
3	0	+	0	+	+	0	+	+	+	+	0	+	0	+	+	0	+	0	0	0	+	0	+	1+	1+
4	0	0	+	+	+	0	+	0	+	+	+	0	0	+	0	+	+	+	0	0	+	0	+	2+	1+
5	0	0	0	+	+	+	+	+	0	+	0	+	0	+	+	+	+	0	0	0	+	0	+	w	0
6	+	0	0	+	+	0	+	+	0	+	+	+	0	0	+	+	0	+	0	0	+	0	+	w	0
7	+	+	0	0	+	0	+	+	+	0	+	0	0	+	+	+	+	+	+	0	+	+	+	1+	1+
8	0	0	0	+	+	0	+	0	+	0	+	+	+	0	0	+	0	+	0	0	+	0	+	2+	1+
9	+	+	+	+	+	0	+	+	+	0	+	0	0	+	+	+	+	+	0	+	+	0	+	1+	w
10	+	+	0	0	+	0	+	+	0	0	+	0	+	0	+	0	0	+	0	0	+	0	+	w	0
11	0	0	0	+	+	0	+	+	+	0	+	+	+	0	0	+	+	0	0	0	+	+	+	1+	1+

The irregular antibody identification tests were performed using immediate spin techniques with panel cells (REAGENS).

The reason why the patient developed hemolytic anemia and generated suspected anti-N during this hospitalization remained unexplained. Considering that the patient had no history of blood transfusion during this period, had received a large amount of vancomycin, which demonstrated a temporal relationship with the hemolysis, and that no other likely cause of hemolytic anemia was identified, we assessed the presence of vancomycin-dependent antibodies in the patient’s serum.

#### 2.1.1 Detection of vancomycin-dependent antibody

In the first step, we prepared a vancomycin solution at a concentration of 1 mg/mL in phosphate-buffered saline (PBS). Then, adding 50 μL of the vancomycin solution to a mixture containing 100 μL of the patient’s serum and 50 μL of M-N+ RBCs produced visible agglutination after centrifugation, which was much stronger than that observed when an equivalent volume of PBS was added (Table 3). We also conducted control experiments by replacing the patient’s serum with normal AB-type serum, which tested negative in antibody screening, and observed no agglutination when either the vancomycin solution or PBS was added. Further tests using RBCs with different expressions of blood group N antigen revealed a direct correlation between the expression of blood group N antigen and the intensity of agglutination (Table 3). This increase in agglutination intensity with higher expression of blood group N antigen suggested that antibody reactivity showed N antigen specificity and dose dependency. The antibody titer was 32 when tested using M-N+ RBCs with the addition of vancomycin solution, whereas the titer was 8 with M+N- RBCs under the same conditions (Table 3).

**Table 3:** Results of vancomycin-dependent antibody tests.

Groups	Agglutination intensity	Antibody titer
The patient’s serum + M-N+ RBCs + vancomycin	4+	32
The patient’s serum + M-N+ RBCs + PBS	2+	NT
Normal AB type serum + M-N+ RBCs + vancomycin	0	NT
Normal AB type serum + M-N+ RBCs + PBS	0	NT
The patient’s serum + M+N+ RBCs + vancomycin	3+	NT
The patient’s serum + M+N+ RBCs + PBS	1+	NT
The patient’s serum + M+N- RBCs + vancomycin	2+	8
The patient’s serum + M+N- RBCs + PBS	0	NT
The patient’s serum + vancomycin-treated M+N- RBCs	0	NT

The vancomycin-dependent antibody tests were performed using immediate spin techniques with antibody screening cells (Shanghai Hemo Pharmaceutical & Biological Co., Ltd). NT, not tested.

Next, we prepared vancomycin-treated M+N- RBCs by mixing the RBCs and vancomycin solution in equal volume at 37 °C for 30 min, followed by three washes with saline to remove unbound drug molecules. However, the patient’s serum exhibited no reactivity to these vancomycin-treated RBCs.

Based on these observations, we concluded that vancomycin-induced antibodies can mediate RBC agglutination in the presence of soluble drug and demonstrate greater specificity for the N blood group antigen.

The patient’s platelet counts remained within normal limits during his hospitalization. To investigate whether the induced antibody could bind to platelets and cause immune-mediated destruction of platelets, antiplatelet antibody tests were performed. The patient’s serum showed weak binding to platelets, but the addition of vancomycin solution did not enhance this reactivity.

## 3 Discussion

DIIHA may be a fatal adverse reaction in some cases, primarily caused by the existence of drug-induced antibodies. Patients with DIIHA may have signs of the rapid destruction of RBCs inside blood vessels shortly after drug administration. Antibodies associated with DIIHA include two main types: drug-dependent antibodies and drug-independent antibodies. Drug-dependent antibodies are the more common forms, which can be classified into two major subtypes, defined by *in vitro* activity. One type of antibody can react with RBCs that are coated with drugs, which generally bind to the RBC membrane covalently, and the drug coating will remain on the RBCs after several washes *in vitro*. The other situation is that drugs do not bind to the RBCs covalently, and drug-coated RBCs cannot be prepared as a target to detect antibodies. The antibodies can be detected by incubating them with soluble drugs and compatible RBCs. Drug-independent antibodies appear to be RBC autoantibodies, instead of antibodies to the drug. It is thought that drugs evoking these antibodies do it by having a direct effect on the immune system. In addition, some drugs cause DIIHA with no antibodies involved. These drugs can change the RBC membrane so that proteins attach to the RBCs and RBCs are destroyed by macrophages in the spleen and liver, which is termed “non-immunological protein adsorption (NIPA)” (12–14). Moreover, some drugs show characteristics of more than one mechanism.

The majority of DIIHAs display positive results in DATs with anti-IgG and anti-C3 (15), such as this case, reflecting severe hemolytic anemia driven by immune complexes, indicating persistent intravascular and extravascular hemolysis. In this case, the patient exhibited severe symptoms of hemolysis, including reduced hemoglobin, raised indirect bilirubin and lactate dehydrogenase, and positive urinary occult blood, after repeated exposure to vancomycin. The detection of vancomycin-dependent antibodies provided the most direct evidence for clinical diagnosis. A definitive de-challenge response could not be assessed due to the patient’s unfortunate death shortly after drug discontinuation. Other potential etiologies for hemolysis, including underlying hematological diseases, autoimmune disorders, infections, and mechanical causes, were systematically investigated and excluded. The causal relationship between vancomycin administration and the onset of hemolysis in this case was assessed as “Probable” according to the WHO-Uppsala Monitoring Center criteria (16). The patient had a serum antibody that reacted with untreated RBCs in the presence of vancomycin, while the antibody was non-reactive with RBCs previously treated with vancomycin. These findings support the hypothesis that vancomycin does not covalently bind to the RBC surface and that the antibody-RBC interaction depends on the presence of soluble vancomycin. The patient’s serum and acid eluate, in our case, produced positive results in antibody screening tests, probably because there were still residual drugs in the patient’s serum, and the vancomycin-dependent antibodies had bound to the RBCs to form immune complexes. While our laboratory investigation conclusively identified vancomycin-dependent antibodies, we acknowledge that potential hemolytic effects from other concomitant medications were not systematically excluded by antibody testing. However, given the compelling temporal relationship and the specificity of the serological confirmation for vancomycin, it remains the most strongly implicated agent in this case of DIIHA.

It was notable that the reaction pattern between the patient’s serum and reagent RBCs with different antigens showed different intensities, suggesting the possible presence of irregular antibodies, likely anti-N, which can lead to ineffective transfusions and hemolysis. However, the patient’s antibody screening tests prior to the development of hemolytic anemia were all negative, and he had no history of blood transfusion during this hospitalization. Given that the patient’s MN blood type was identified as M-N+, the anti-N was likely an autoantibody. Thus, we investigated and confirmed that hemolytic anemia was caused by drug-dependent antibodies and that these antibodies were specific to the blood group N antigen. To our knowledge, this is the first reported case demonstrating a vancomycin-dependent antibody with novel specificity for the blood group N antigen. Some drug-dependent antibodies can show specificity for certain blood group antigens. For instance, a previous study reported a 50-year-old woman exposed to piperacillin who developed autoantibodies with blood group e antigen specificity on the 12th day of antibiotic treatment (17). These results suggest that irregular antibodies may delay the diagnosis of drug-dependent antibodies, and that the assessment of drug-dependent antibodies should also consider the evaluation of their blood group specificity.

The vancomycin-dependent antibodies in the patient’s serum appeared to be primarily IgG, indicating that there may be a transition from IgM antibodies to IgG antibodies during the immune response in this case. Unfortunately, daily monitoring was not performed, so complete tracking of serological changes and disease progression was lacking.

Furthermore, drug-dependent antibodies can also target platelets, resulting in thrombocytopenia (18). In our patient, platelet counts remained within normal limits, and antiplatelet antibody tests were only weakly positive, indicating that vancomycin primarily caused immune hemolysis of RBCs, with minimal effect on platelets.

In summary, this case illustrates the complexity of testing for DIIHA and the importance of clinical recognition. DIIHA should be considered in patients with new-onset hemolytic anemia of unclear cause who have recently received medications, especially antibiotics. Timely serological testing and immediate discontinuation of the causative drug remain the most effective strategies to prevent the serious consequences of DIIHA.

## Data Availability

The original contributions presented in the study are included in the article/supplementary material, further inquiries can be directed to the corresponding authors.
